# Safety and effectiveness of dual channels vancomycin administration in the treatment of intracranial infection after severe brain injury surgery

**DOI:** 10.1097/MD.0000000000039410

**Published:** 2024-09-20

**Authors:** Ao Jiao, Wanjiang Hao, He Yang, Yanli Du

**Affiliations:** a Medical College of Soochow University, Suzhou, Jiangsu, China; b Hulun Buir People’s Hospital, Hulunbuir, China.

**Keywords:** adverse reactions, cerebrospinal fluid, intracranial infection, severe craniocerebral injury, vancomycin

## Abstract

To observe the clinical efficacy and safety of vancomycin intravenous drip combined with vancomycin intrathecal injection in the treatment of intracranial infection after severe brain injury surgery. From January 2020 to June 2022, 80 patients with intracranial infection after severe brain injury surgery were selected and randomly divided into 2 subgroups; there were 40 patients in each subgroup. All patients were treated with vancomycin. The control subgroup was medicated with intravenous drip, and the observation subgroup was treated through 2 channels (intravenous drip + intrathecal injection), with a course of 7 days. The clinical efficacy, intracranial pressure, infection control time, routine indexes of cerebrospinal fluid (white blood cell count [WBC], glucose content [Glu], and total protein content [Pro]) and the incidence of adverse reactions were contrasted between the 2 subgroups. Versus the control subgroup, the total effective rate in the observation subgroup was notably higher (95.00% vs 77.50%). After treatment, aiming at the intracranial pressure and infection control time, versus the control subgroup (146.20 ± 22.37) mmH2O and (9.86 ± 1.62) days, the observation subgroup were (125.43 ± 18.5) mmH2O and (7.35 ± 1.57) days respectively, which were notably lower. After treatment, versus the control subgroup, the concentrations of WBC and Pro in cerebrospinal fluid in the observation subgroup were lower, and the content of Glu was higher. There was no statistical distinction in the incidence of adverse reactions between the 2 subgroups (17.50% vs 10.00%). Two-channel administration of vancomycin can improve the clinical efficacy of internal infection after severe craniocerebral injury, reduce intracranial pressure, and cerebrospinal fluid WBC and Pro levels, and has high safety.

## 1. Introduction

**Figure 1.**
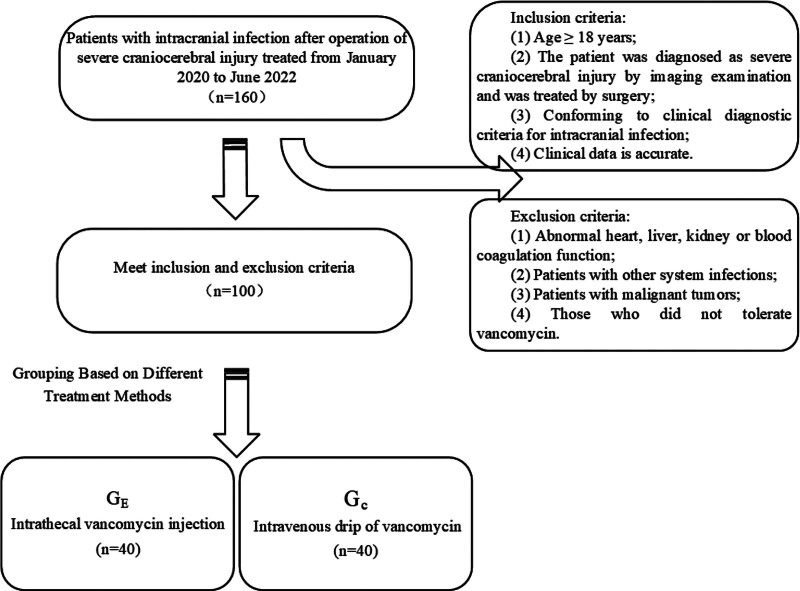
Dual channels vancomycin administration in the treatment of intracranial infection after severe brain injury surgery.

Intracranial infections are a common and serious complication following major cranial surgeries, characterized by a high mortality rate and a high rate of disability.^[1]^ This is primarily attributed to the complexity and extended duration of cranial surgeries, which increase the susceptibility to intracranial infections. Furthermore, the unique physiological structure of the brain poses significant challenges in controlling these infections.^[2,3]^ Clinical analysis shows that the main pathogens of intracranial infection after craniocerebral injury surgery are gram-positive bacteria, such as Staphylococcus aureus, methicillin-resistant Staphylococcus aureus, etc.^[4]^ Vancomycin is the first choice for clinical treatment of intracranial infection. In the past, large doses of intravenous administration were often adopted. However, due to the influence of the blood-brain barrier, few drugs entered the cerebrospinal fluid, and the efficacy was not ideal.^[5,6]^ Intrathecal administration does not pass through the blood-brain barrier, which can increase the local drug concentration of cerebrospinal fluid, and has a definite effect and quick onset.^[7]^ In view of this, the authors discussed the efficacy and safety of intravenous infusion of vancomycin plus intrathecal injection dual channels administration in the treatment of intracranial infection after severe craniocerebral injury; see Figure 1 for the specific scheme.

## 2. Data and methods

### 2.1. General information

The study was approved by the Ethics Committee of Soochow University School of Medicine. The subjects were patients with intracranial infection after operation of severe craniocerebral injury treated from January 2020 to June 2022 in our hospital. The researchers prepared 80 envelopes in advance, with each envelope containing group assignment information for either the control group or the experimental group, with 40 envelopes for each group. These envelopes were sealed and placed in a box. When eligible patients were enrolled in the study, a random envelope was drawn to assign the patient to either the control or experimental group, with 40 patients in each subgroup. The observation subgroup comprised 25 males and 15 females, with an average age of (53.18 ± 10.46) years, ranging from 24 to 72 years old. Twenty instances were injured by traffic accidents, 13 instances were injured by falling from high places, and 7 instances were injured by blows. The control subgroup comprised 27 males and 13 females, with an average age of (54.27 ± 10.55) years, ranging from 22 to 75 years old. There were 18 instances of traffic accident injuries, 12 instances of falling injuries, and 10 instances of hitting injuries. There was no statistical distinction in general data between the 2 subgroups (*P* > .05), which was comparable. This study has been approved by the medical ethics committee of the hospital, and all patients or family members have informed consent to the study content. The formula for calculating the sample size is as follows:


$$\begin{array}{l} n=\frac{{\left(Z_{\alpha }+Z_{\beta }\right)}^{2}\cdot \left(\sigma_{1}^{2}+\sigma_{2}^{2}\right)}{d^{2}} \end{array}$$

1) The letter “n” represents the sample size in each group.
2) *Z*_α_ represents the *Z* score at significance level α. For a 2-tailed test, α is typically set at 0.05, so *Z*_α_ is 1.96.
3) *Z*_β_ is the *Z*-score corresponding to statistical power (1−β). With a statistical power of 0.8, *Z*_β_ is about 0.84.
4) σ_1_ and σ_2_ represent the standard deviations of the 2 groups, which, in this case, are both 30.
5) *d* represents the effect size, which is the difference between the means of the 2 groups, in this case, it is 20.

### 2.2. Inclusion and exclusion criteria

Inclusive criteria: Age ≥ 18 years; The patient was diagnosed as severe craniocerebral injury by imaging examination and was treated by surgery; Conforming to clinical diagnostic criteria for intracranial infection^[8]^; Clinical data is accurate. Exclusion criteria: Abnormal heart, liver, kidney or blood coagulation function; Patients with other system infections; Patients with malignant tumors; Those who did not tolerate vancomycin.

### 2.3. Treatment methods

All patients were treated with lowering intracranial pressure, dehydration, nourishing nerves, etc. On this basis, the control subgroup received intravenous drip of vancomycin (GYZZ H20084269, Zhejiang Haizheng Pharmaceutical Co., Ltd., Taizhou, China, 1.0 g/bottle), 1000 mg/time, 12 hour/time. The observation subgroup was treated with intravenous drip combined with intrathecal administration. The dosage of intravenous drip was the same as that of the control subgroup. The intrathecal administration was punctured through the lumbar spine, and diluted vancomycin was injected into the subarachnoid space; the dose was 0.3 mg/kg, once a day. Both subgroups were treated continuously for 7 days.

### 2.4. Observation indicators

Intracranial pressure and infection control time: Intracranial pressure was measured before and after treatment, and infection control time was recorded. Routine index of cerebrospinal fluid: Before and after treatment, 2 mL of patients’ cerebrospinal fluid was collected to detect the white blood cell count (WBC), glucose content (Glu), and total protein content (Pro). Adverse reactions: The adverse reactions related to drugs occurred during the treatment were counted. The assessment of all indicators was carried out by independent professionals who were blinded to the patient’s group allocation, employing an assessor-blinded approach.

### 2.5. Efficacy determination

Recovery: After treatment, the patient’s clinical symptoms and signs completely disappeared, the cerebrospinal fluid examination items were normal, and the bacteriological test showed negative.
Notable effect: After treatment, the patient’s clinical symptoms and signs were notably improved, and there was at least 1 abnormal cerebrospinal fluid examination or bacteriological test.
Effective: After treatment, the clinical symptoms and signs of the patients were improved, but the cerebrospinal fluid examination and bacteriological examination were abnormal.
Ineffective: After treatment, the symptoms and signs of the patients were not improved or aggravated.
Total effective rate = (recovery + notable effect + effective) number of instances/total number of instances × 100%.
Neurological symptoms and signs: The assessment was conducted using the symptom and sign items within the NIHSS scoring system. A score of 0 indicates complete absence of symptoms and signs. A decrease in NIHSS score of ≥10 points after treatment was considered a “notable effect,” a decrease of ≥5 points was considered “effective,” and all other cases were categorized as “ineffective.”
Normal range for cerebrospinal fluid parameters: Cerebrospinal fluid (CSF) pressure within the range of 70 to 180 mmH2O; CSF white blood cell count between 0 and 5 cells/μL; CSF glucose concentration between 50 and 75 mg/dL; CSF total protein concentration between 15 and 45 mg/dL.
Bacteriological examination normal range: Negative results in bacterial culture.

### 2.6. Data statistics

The data were analyzed by SPSS 22.0 software, and the measurement data (in accordance with normal distribution and uniform variance) and counting data were analyzed by (*x* ± *s*) and [n (%)], respectively. *t* value and χ^2^ value test were used, and *P* < .05 was regarded as statistical distinction.

## 3. Results

### 3.1. Clinical efficacy

**Figure 2.**
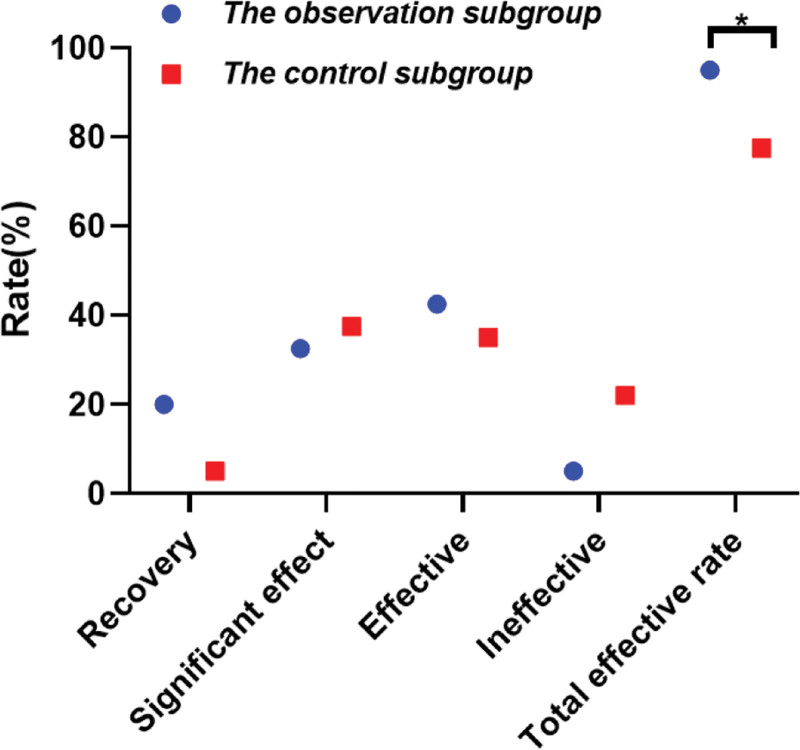
Clinical efficacy the 2 subgroups. *P < .05.

Versus the control subgroup (77.50%), the total effective rate of the observation subgroup was 95.00%, which was notably higher (*P* = .023 < .05), as displayed in Table 1 and Figure 2. In this context, the overall incidence rate of adverse reactions is calculated once for study subjects with multiple adverse events, without duplication.

**Table 1:** Distinction of clinical efficacy between the 2 subgroups [n (%)].

Project	n	Recovery	Notable effect	Effective	Ineffective	Total effective rate
The observation subgroup	40	8 (20.00)	13 (32.50)	17 (42.50)	2 (5.00)	38 (95.00)
The control subgroup	40	2 (5.00)	15 (37.50)	14 (35.00)	9 (22.00)	31 (77.50)
χ^2^ value						5.165
*P* value						.023

### 3.2. Intracranial pressure and infection control time

**Figure 3.**
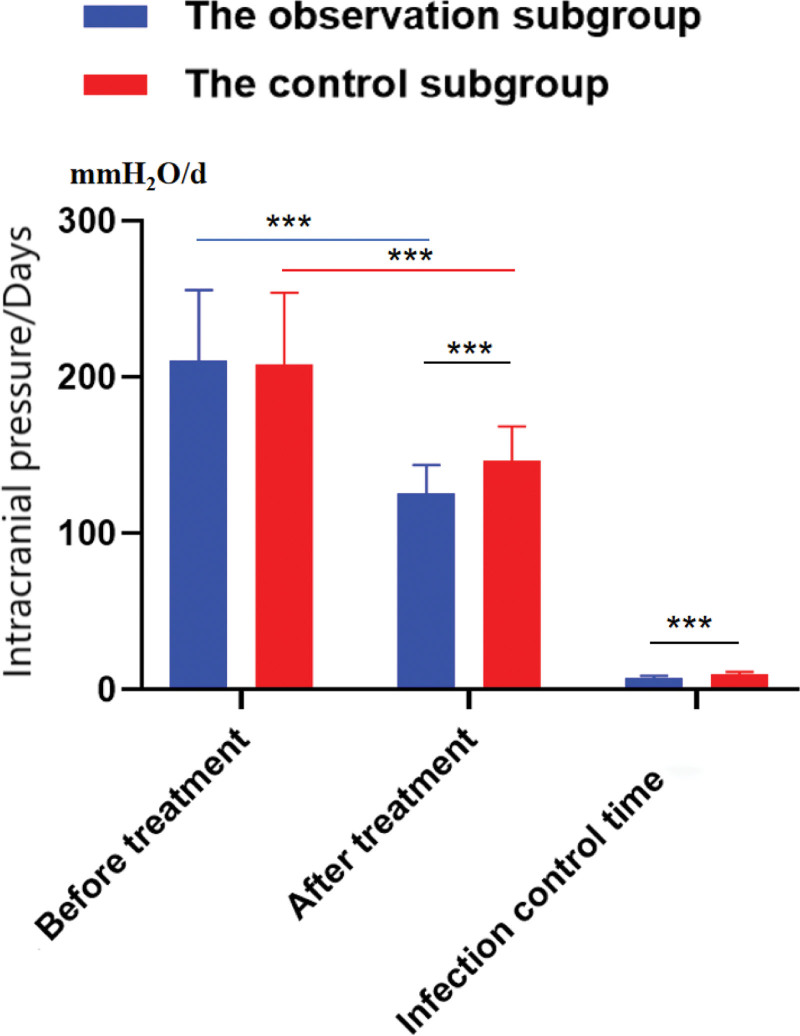
Intracranial pressure and infection control time. *P < .05, versus the observation subgroup before treatment. **P < .05, versus the control subgroup before treatment. ***P < .05, versus the observation subgroup after treatment.

After treatment, the intracranial pressure in the 2 subgroups was notably lower than that before treatment (*P* < .001), and the decrease in the observation subgroup was more notable than that in the control subgroup (*P* < .001). The infection control time in the observation subgroup was (7.35 ± 1.57) days, which was notably less than that in the control subgroup (9.86 ± 1.62) days (*P* < .001), as displayed in Table 2 and Figure 3. The 2 intracranial pressure measurements were conducted on the first day following surgery (prior to treatment initiation) and on the 8th day after the 7-day medication course, in order to eliminate the influence of other procedures.

**Table 2:** Distinction of intracranial pressure and infection control time between the 2 subgroups (x̄ ± *s*).

Project	n	Intracranial pressure (mmH_2_O)		Infection control time (d)
		Before treatment	After treatment	
The observation subgroup	40	210.56 ± 45.28	125.43 ± 18.50*	7.35 ± 1.57*
The control subgroup	40	207.81 ± 46.35	146.20 ± 22.37*	9.86 ± 1.62*
*t* value		0.268	4.525	7.037
*P* value		.789	<.001	<.001

* *P* < .05, versus the same subgroup before treatment.

### 3.3. Routine indicators of cerebrospinal fluid

After treatment, the levels of WBC and Pro in cerebrospinal fluid of the 2 subgroups were notably lower than those before treatment (*P* < .001), while the level of Glu was notably higher than that before treatment (*P* = .007 < .05). The changes of the above indexes in the observation subgroup were more notable (*P* < .001), as displayed in Table 3.

**Table 3:** Distinction of routine indexes of cerebrospinal fluid between the 2 subgroups before and after treatment (x̄ ± s).

Project	n	WBC (×10^6^/L)		Glu (mmol/L)		Pro (g/L)	
		Before treatment	After treatment	Before treatment	After treatment	Before treatment	After treatment
The observation subgroup	40	12.85 ± 3.25	5.14 ± 1.22	2.37 ± 0.68	4.15 ± 1.54*	1.61 ± 0.48	0.55 ± 0.12*
The control subgroup	40	12.47 ± 3.18	7.58 ± 1.57*	2.40 ± 0.65	3.26 ± 1.31*	1.65 ± 0.51	0.89 ± 0.23*
*t* value		0.529	7.761	0.202	2.784	0.361	8.289
*P* value		.599	<.001	.841	.007	.719	<.001

* *P* < .05, versus the same subgroup before treatment.

### 3.4. Adverse reactions

**Figure 4.**
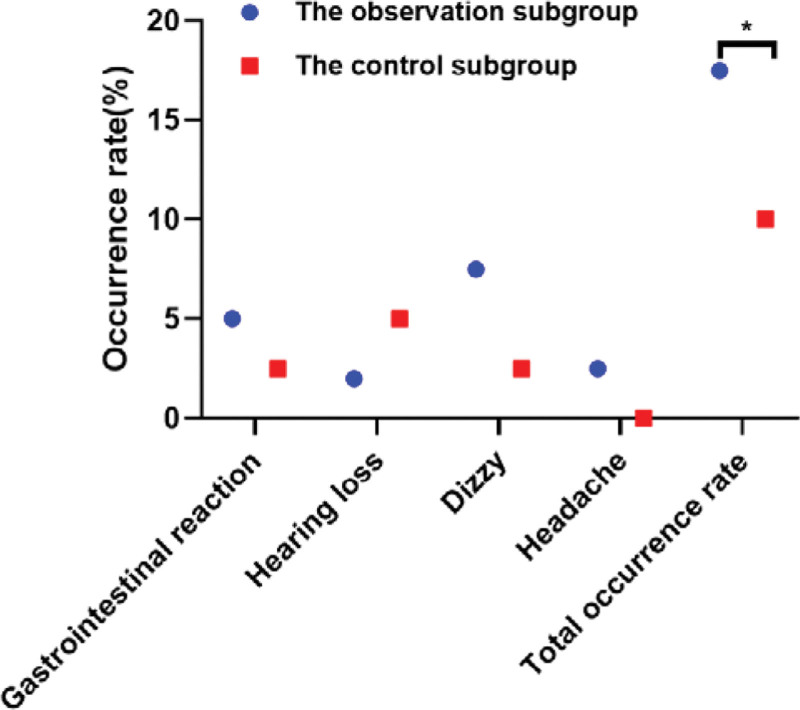
Adverse reactions. *P < .05.

No serious adverse reactions occurred in the 2 subgroups during the treatment period, in which the incidence of adverse reactions in the observation subgroup was 17.50%, compared with 10.00% in the control subgroup, the distinction was not statistically notable (*P* = .33 > .05), as displayed in Table 4 and Figure 4.

**Table 4:** Distinction of adverse reactions between the 2 subgroups [n (%)].

Project	n	Gastrointestinal reaction	Hearing loss	Dizzy	Headache	Total occurrence rate
The observation subgroup	40	2 (5.00)	1 (2.50)	3 (7.50)	1 (2.50)	7 (17.50)
The control subgroup	40	1 (2.50)	2 (5.00)	1 (2.50)	0 (0.00)	4 (10.00)
χ^2^ value						0.949
*P* value						.330

## 4. Discussion

According to the survey,^[9]^ the incidence of intracranial infection after craniocerebral injury surgery is 2.6%~5.0%, which is one of the main risk factors leading to death. Intracranial infection usually occurs within 3~7 days after craniotomy. The main clinical manifestations are increased intracranial pressure, brain edema, coma, epilepsy, etc. If treatment is not timely, it may lead to paralysis, intellectual impairment, death and other serious consequences.^[10–13]^ Clinically, intracranial infections are mostly caused by gram-positive bacteria, mainly staphylococci. With the widespread use of antibiotics, intracranial infections caused by methicillin-resistant Staphylococcus aureus are increasing year by year.^[1,14]^ For intracranial infection, doctors generally use vancomycin empirically, and the common way of administration is intravenous drip, but it is found that long-term high-dose intravenous infusion of vancomycin can not effectively control intracranial infection, but also damage the liver and kidney function of patients.^[15–17]^ This is mainly affected by the obstruction of the blood-brain barrier, resulting in the cerebrospinal fluid vancomycin cannot reach the effective bacteriostatic concentration. Through the continuous attempts of clinicians, it is found that intrathecal administration, that is, the drug directly into the subarachnoid space, without passing through the blood-brain barrier, can increase the local drug concentration and avoid the side effects caused by long-term intravenous drip, but there are also certain risks. It has not been widely used in clinic.^[18]^

The results of this study showed that the total effective rate of the observation subgroup was higher than that of the control subgroup (95.00% vs 77.50%), indicating that dual channels administration of vancomycin can improve the therapeutic effect of intracranial infection. Vancomycin is the first choice for clinical treatment of intracranial infection, and it is highly sensitive to staphylococci. Its mechanism of action is: Act on the precursor of the cell front wall D-alanine-D-alanine, block the synthesis of peptidoglycan, interfere with the formation of the cell wall, thus blocking the growth and reproduction of bacteria.^[19,20]^ Due to the existence of the blood brain barrier, the drugs injected intravenously into the cerebrospinal fluid are insufficient and cannot reach the effective inhibitory concentration, so the effect of intravenous drip alone is not good. The injection of vancomycin through subarachnoid space can make the drug directly reach the infection site, and improve the local drug concentration, with notable effect. Previous studies^[21,22]^ have shown that compared with intravenous drip, intrathecal vancomycin is more effective in treating postoperative intracranial infection. Considering that the increase of bacterial resistance and the operation may cause the interruption of cerebrospinal fluid circulation and prevent the local intrathecal injection of drugs from entering the infection site, this study uses intravenous drip combined with intrathecal administration of drugs to further improve the clinical efficacy of intracranial infection after severe brain injury surgery. The article also showed that the intracranial pressure of the observation subgroup was lower than that of the control subgroup, and the infection control time was shorter than that of the control subgroup, which further explained that the dual channels administration of vancomycin could improve the therapeutic effect of intracranial infection. The study by Zhang et al^[23]^ concluded that intrathecal injection of meropenem and vancomycin is more effective than intravenous injection in the treatment of intracranial infection after craniotomy. And our research on this basis, The control subgroup was medicated with intravenous drip, and the observation subgroup was treated through 2 channels (intravenous drip + intrathecal injection), with a course of 7 days. Our study demonstrates that 2-channel administration of vancomycin can improve the clinical efficacy of internal infection after severe craniocerebral injury. Of course, Similar conclusions were reached between our 2 studies, demonstrating the efficacy and safety of intrathecal use.

In patients with intracranial infection, WBC and Pro may increase in the early stage. With the improvement of the disease, the levels of WBC and Pro will decrease notably until they return to normal, which has certain predictive value for the prognosis of patients.^[24,25]^ The related report^[26]^ shows that Glu is the main energy supplying substance of the human body, and the brain consumes about 120 g of Glu every day. Its entry into the brain mainly depends on the glucose transporter in the blood-brain barrier. Due to the influence of surgical trauma and infection, the metabolic function of the body is abnormal and the amount of Glu entering the cerebrospinal fluid decreases. The results of this study showed that after treatment, the levels of WBC and Pro in cerebrospinal fluid in the observation subgroup were notably lower than those in the control subgroup, while the level of Glu in the observation subgroup was notably higher than that in the control subgroup, indicating that dual channels administration of vancomycin can effectively improve the condition of patients with intracranial infection after severe craniocerebral injury. This is because of the dual channels administration, vancomycin is distributed in both of blood and cerebrospinal fluid of the patients, the drug concentration is high, and the condition recovers quickly. It should be noted that while dual channels administration increases the drug concentration, it may also induce adverse reactions due to high local concentration.^[27]^ In this study, it was found that there was no notable distinction in the incidence of adverse reactions between the 2 subgroups, indicating that dual channels administration of vancomycin is safe in the treatment of intracranial infection after severe craniocerebral injury. Intrathecal administration of vancomycin belongs to local application, but the volume of the ventricle is small, and the toxic reaction is large, which may lead to subarachnoid adhesion, nerve stimulation and so on. In severe instances, it can also lead to coma or death.^[5,28,29]^ At present, there is no dose standard for vancomycin intrathecal administration in clinical practice.

## 5. Conclusion

Intravenous drip combined with intrathecal administration is safe and effective for the treatment of intracranial infection after severe brain injury surgery, which can be used as one of the treatment schemes for intracranial infection.

## Author contributions

**Conceptualization:** Ao Jiao.

**Data curation:** Ao Jiao, Wanjiang Hao.

**Formal analysis:** Wanjiang Hao, He Yang.

**Investigation:** He Yang, Yanli Du.

**Methodology:** He Yang, Yanli Du.

**Supervision:** Wanjiang Hao, Yanli Du.

**Writing – original draft:** Ao Jiao.

**Writing – review & editing:** Ao Jiao, Yanli Du.
